# Haemophilia A: Pharmacoeconomic Review of Prophylaxis Treatment versus On-Demand

**DOI:** 10.1155/2015/596164

**Published:** 2015-01-05

**Authors:** Brigid Unim, Maria Assunta Veneziano, Antonio Boccia, Walter Ricciardi, Giuseppe La Torre

**Affiliations:** ^1^Department of Public Health and Infectious Diseases, Sapienza University of Rome, Piazzale Aldo Moro 5, 00185 Rome, Italy; ^2^Institute of Hygiene, Catholic University of the Sacred Heart, Largo Agostino Gemelli 8, 00168 Rome, Italy

## Abstract

*Objectives*. Haemophilia A is a congenital disorder of coagulation that mainly affects males and causes a considerable use of resources, especially when hemophilic patients are treated with prophylaxis. The aim of the present review was to discuss and appraise the methodological aspects and results of published economic evaluations of haemophilia A treatments in the last decade. *Methods*. The literature search, performed by consulting four engines, covered studies published between 2002 and 2014. Full economic evaluations published in English language were identified and included in the review. A quality assessment of the studies was also carried out based on Drummond's checklist. *Results*. After careful evaluations of the identified records, 5 studies were reviewed. Primary and secondary prophylaxis resulted cost-effective compared to on-demand therapy: the ICER of primary prophylaxis ranged from €40.236 to €59.315/QALY gained, while the ICER of secondary prophylaxis was €40.229/QALY gained. Furthermore, 60% were high quality and 40% were medium quality studies. *Conclusions*. The review underlines the cost-effectiveness of prophylaxis versus on-demand treatment and the different methodological approaches applied. Further economic evaluations are required with models that reflect the clinical reality and consumption of resources in each country.

## 1. Introduction

Haemophilia A is a congenital disorder of coagulation that mainly affects males; the prevalence in the Italian population was 6.2/100.000 inhabitants in 2012 (95%CI 6.0–6.4) and 12.7/100.000 inhabitants (95%CI 12.3–13.1) among the Italian males [1]. As other chronic diseases, haemophilia has high economic burden, especially when patients are treated with prophylaxis. This is mainly due to the complex therapy management process that involves expensive treatments. In particular, as confirmed by the available scientific literature, haemophilia A is one of the most expensive diseases because of the required lifetime treatment and the management of the related adverse effects and complications, especially when patients develop factor VIII inhibitors [2–5]. In addition, indirect costs, usually expressed in terms of considerable loss of productivity for the society, should be taken into account [6, 7]. High healthcare costs are due to the continuous factor VIII (FVIII) infusions therapy that people affected by haemophilia A need for the management and the prevention of bleedings and to reduce the risk of complications, such as flexion contractures, joint arthritis/arthropathy, chronic pain, muscle atrophy, compartment syndrome, neurologic impairment.

Replacement therapy is the main treatment for haemophilia A; it consists in infusions aimed at replacing the missing or faulty clotting factor in the blood stream. In particular, infusions could be injected either on-demand or on regular basis (prophylaxis). According to the scientific literature, prophylaxis is the first choice therapy, especially for children with severe haemophilia A [8].

Several economic evaluations have been conducted to compare on-demand versus prophylactic treatment in terms of cost-effectiveness and/or cost-utility.

The current paper is a systematic review of full economic evaluations (cost-effectiveness and cost-utility analyses), conducted in different countries. The objective of this review was to discuss and appraise the methodological aspects and results of published economic evaluations of haemophilia A available treatments from January 2002 to November 2014.

## 2. Materials and Methods

### 2.1. Identification of Relevant Studies

The literature search, which covered studies published from 1st January 2002 until 3rd November 2014, was performed by consulting PubMed and Scopus engines. Further research was carried out through Google and Google scholar. Search terms were used as follows:

1. (cost effectiveness) AND (haemophilia prophylaxis versus on-demand);
2. (cost-utility) AND (haemophilia prophylaxis versus on-demand).

**Figure 1:**
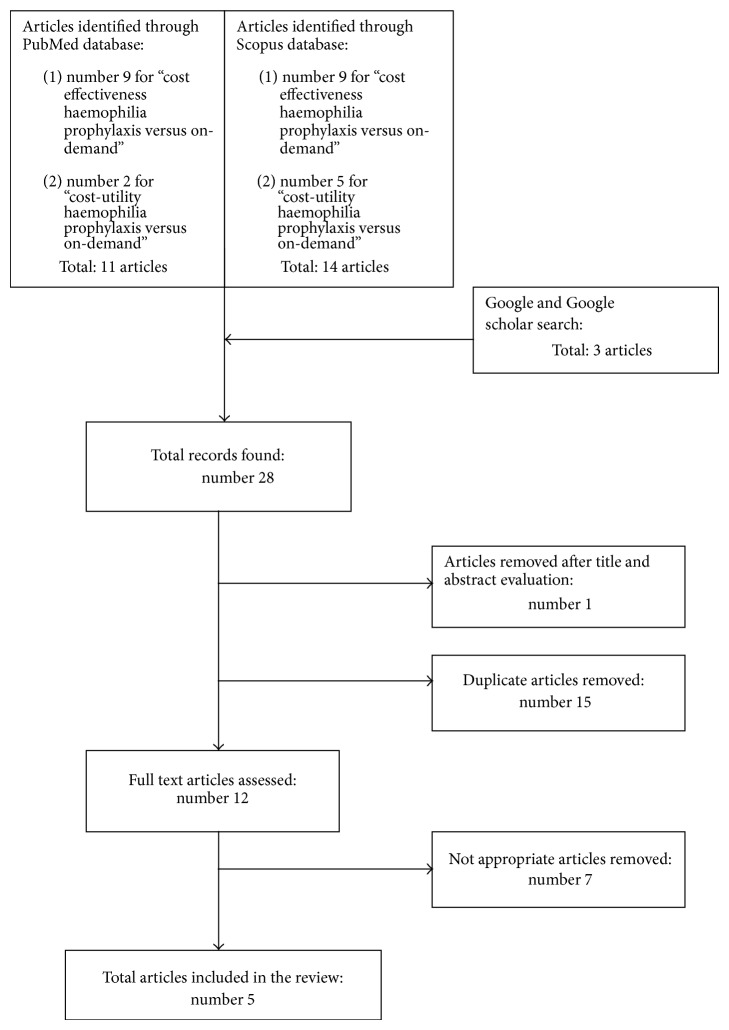
Flow chart of the selection process.

The only limit set for all search engines was the publication date. Search criteria are summarized in Figure 1. In the selection process, abstracts were initially read independently by two researchers to identify the potentially eligible full text papers, which were then retrieved and assessed in order to decide on the final inclusion. Full economic evaluations (cost-effectiveness and cost-utility analysis) published in English language were identified and included in the review.

Full economic evaluation is the comparative analysis of alternative courses of action in terms of both costs (resource use) and consequences (outcomes, effects) [9]. Studies on the use of healthcare resources that do not make explicit comparisons between alternative interventions in terms of both costs (resource use) and consequences (effects) are considered as partial economic evaluations and include cost analyses, cost-description studies, and cost-outcome descriptions [10].

Consequently, partial economic evaluations that did not meet the inclusion criteria above explained were excluded. Reviews, duplicate articles, and studies without available full text were also eliminated.

For comparison of results on haemophilia treatment, studies considering inhibitor patients were also excluded since the presence of inhibitors requires different treatment modality associated with higher health care cost and reduced overall efficacy compared to replacement of the deficient factor. Furthermore, all costs are express in Euro and, when necessary, they were converted to Euro by using the Euro foreign exchange reference rates as at November 2014.

### 2.2. Quality Assessment and Data Extraction

A quality assessment of the included studies was carried out, based on the British Medical Journal (BMJ) referees' checklist proposed by Drummond and Jefferson [11] and modified by La Torre et al., weighting-median score for each item by different experts [12].

Drummond's checklist is composed of 35 items divided into 3 sections: study design, data collection and analysis, and interpretation of results. To weight the items, a group of experts attributed a score according to their importance. The weighted scores assigned by the consensus to study design, data collection and analysis, and interpretation of results were 26, 45, and 48, respectively (total score = 119). For each item section, the maximum achievable score was as follows:

1. study design (7 items), maximum global score = 26;
2. data collection (14 items), maximum global score = 45;
3. analysis and interpretation of results (14 items), maximum global score = 48.

When the item was not applicable to the study, we reduced the maximum global score from the relative weighted score item.

Discrepancies between the two investigators were solved by oral discussion and consensus with senior investigators (GLT, WR). Each item was assigned with the median weight attributed by the consensus, if applicable. Finally, we obtained the global score summing up weights of each item. Studies achieving a score above 90 were considered of high quality.

Two reviewers used a data collection form to independently abstract data from the studies. The information extracted is the following: reference with publication year, type of analyses, alternatives, country/perspective, patients/time horizon, effectiveness measurement/cost measurement, and results. The reviewers discussed any discrepancies in their results to reach an agreement. The characteristics of each study are depicted in Table 1.

The review results are presented according to the PRISMA (Preferred Reporting Items for Systematic Reviews and Meta-Analyses) statement [13].

## 3. Results

### 3.1. Literature Search

The literature search, using the two strings above mentioned, brought out 28 potentially useful articles:

- 11 for PubMed search;
- 14 for Scopus search;
- 3 additional records for Google and Google scholar search.

The articles were screened based on title and abstract and, after the removal of duplicate records and nonrelevant articles, five studies were included in the review. The excluded studies were 15 duplicate records, 1 review article, 2 articles considering haemophilia patients with inhibitors, and 5 partial economic analyses (Figure 1).

Most economic evaluations included in the review compared costs and clinical outcomes associated with the infusion of FVIII in prophylaxis and on-demand treatment. Moreover, all studies included a cost-utility analysis in which quality-adjusted life-years (QALYs) are used as measures of health effects [14–18]. The analyses were conducted mainly in Europe. In particular, two studies have been conducted in the United Kingdom (UK) [14, 15], one in Canada [16], one in Germany/Sweden/UK/the Netherlands [17], and one in Italy [18].

### 3.2. Review Results

The study by Miners and colleagues (2002) was a cost-utility analysis, conducted in UK from the societal perspective [14]. Two treatment options were compared: primary prophylaxis and on-demand treatment. The sample included in the analysis was a hypothetical cohort of patients with severe haemophilia A or haemophilia B and von Willebrand disease. Data on clinical effects and costs of treatments were combined in the Markov model. As the study was conducted from the societal point of view, indirect costs, expressed in terms of school/work days lost, were also considered in addition to direct costs. The analysis showed that primary prophylaxis, compared with on-demand treatment, was cost-effective. The baseline analysis evidenced that the incremental cost-effectiveness ratio (ICER) was *£*46.500 (€59.315) per QALY gained, for individuals receiving FVIII and factor IX (FIX).

In 2009, Miners modified the 2002 model [14], by updating the previously published data [15]. The study was conducted from the perspective of the National Health Service (NHS). In the study published in 2002 [14], only costs were discounted at an annual discount rate of 6%, while in the updated study by Miners [15] an annual discount rate of 3.5% per annum has been applied to both costs and benefits. In this study, there was a gap between the utility gained by patients treated with prophylaxis (0.87) and the utility gained by patient treated on demand (0.66). In the 2009 study, ICER per QALY—in the baseline analysis—was lower (*£*38.000; €48.469) than the value of the study published in 2002 (*£*46.500; €59.315). This difference could depend on the price of FVIII and on the frequency of infusions. However, the sensitivity analysis showed that the results were very sensitive to

- unit cost of clotting FVIII;
- discount rate;
- bleeding frequency;
- utility assumptions.

The study conducted in Canada by Risebrough et al. [16] evaluated the cost-utility profile of escalading-dose (EscDose) compared to on-demand treatment and primary prophylaxis with EscDose. A hypothetical cohort of male patients, aged between 1 and 6 years with severe haemophilia A, was considered. As the analysis was conducted from the societal point of view, indirect costs, expressed in terms of working days lost by parents to assist children, were also included.

A Markov model was developed for the economic analysis, with health states based on the number of “target joints” involved. The results were expressed in terms of Canadian$ (Can$) per QALY. The incremental cost per QALY gained for EscDose compared with primary prophylaxis was more than Can$ 1.000.000 (€698.300 (Exchange rate Can$ 1 = €0.6983 [updated to November 4, 2014])) per QALY gained. On the other hand, by comparing on demand to EscDose, the ICER was Can$ 542.938 (€379.134 (exchange rate Can$ 1 = €0.6983 [updated to November 4, 2014])) per QALY gained.

The sensitivity analysis showed that the results are sensitive to the price of the clotting FVIII and to the number of “target joints” involved. In conclusion, the analysis demonstrated that prophylaxis significantly improved the quality of life of children affected by severe haemophilia A. The study by Lippert et al. [17] was conducted in several European countries (Germany, Sweden, UK, and The Netherlands) from the third-party payers' perspective. A full economic evaluation was performed to estimate the cost-effectiveness of on-demand versus prophylactic therapy. The sample consisted of 516 patients, with severe haemophilia A and haemophilia B, without clotting factor inhibitors (VIII/IX), who aged more than 14 years.

The analysis demonstrated that patients receiving prophylaxis were subject to less bleeding episodes than those who were treated on demand. In this analysis, the results were very different from those of the studies reported above [14–16]. In particular, in the group of patients who aged less than 30 years and were HIV positive, the ICER ranged from €1.2 million/QALY in Germany to €1.72 million/QALY in UK.

On the other hand, in the group of patients who aged less than 30 years and were HIV negative, the ICER was €2.21 million/QALY and €3.10 million/QALY in Germany and in UK, respectively.

Finally, in the group of patients aged more than 30 years old and HIV-negative, ICER ranged from €4.77 million/QALY in Germany to €5.7 million/QALY in Sweden and in the UK.

The study conducted by Colombo et al. [18] was a cost-utility analysis based on the model developed by Miners et al. in 2002 [14]. The Markov model was adapted to the Italian context by replacing some clinical and economic parameters. The analysis was performed from the perspective of the NHS. Four alternatives were compared: primary prophylaxis, secondary prophylaxis, hybrid regimen (primary prophylaxis followed by on-demand treatment), and on-demand treatment. According to Miners' et al. study, published in 2002 [14], a discount rate of 6% was applied to all costs. The analysis showed that the primary and secondary prophylaxes were cost-effective when compared to on-demand treatment (ICER = €40.236/QALY and €40.229/QALY, resp.). The hybrid strategy (prophylaxis therapy followed by on-demand therapy) was less cost-effective than primary and secondary prophylaxes (ICER = €119.134).

Table 1 depicts and summarizes the main characteristics of the studies reviewed.

### 3.3. Results of the Quality Assessment

In all included studies [14–18], the research question (item 1), its economic importance (item 2), the analysis point of view (item 3), and the form of economic evaluation (item 6) were clearly stated. All studies, except one [18], clearly described the rationale for choosing the alternative programmes (item 4), the sources of data effectiveness (item 8), the primary outcomes of the economic evaluation (item 11), and currency and price data (item 18). Moreover, all analysis reported appropriate conclusions (item 34) and caveats (item 35) by giving a satisfactory answer to the study question (item 33). In 3 out of 5 analyses, the choice of the economic evaluation form was justified (item 7) [15, 17, 18], while only one study [16] reported the details of the subjects from whom evaluations were obtained (item 13). All studies reported the methods to evaluate health status and other benefits (item 12).

Despite the discount rates that were stated (item 23) in most studies, except for Lippert et al. [17], only one of them [15] justified the choice of the rates (item 24). In particular, the studies by Colombo et al. [18] and Miners et al., 2002 [14], reported a discount rate of all cost at 6%, whilst Miners 2009 [15] discounted costs at 6% and benefits at 0%. Finally, Risebrough et al. [16] reported a 3% rate for all cost and QUALYs.

All economic evaluations gave details for inflation adjustment or currency conversion (item 19) and model details (item 20). In all studies, relevant alternatives were compared (item 30), incremental analysis was reported (item 31), and outcomes were presented both in aggregate and disaggregate forms (item 32). Most studies (80%) reported costs and resources separately (item 16) and all studies described the methods followed to estimate them (item 17).

Despite the fact that sensitivity analyses were reported in all studies (item 27), only three of them [14–16] justified the choice of the variables included (item 28).

Relative to the scores achieved in each section, in the first section, the quality scores ranged between 19 [18] and 26 [14, 16]; in the second section, the maximum score of 39 was achieved by the study by Risebrough et al. [16] while the minimum score of 28 was reached by Lippert et al. [17]. In the third section, the scores ranged between 32 [17] and 39 [14–16] (Table 2). Therefore, highest scores were achieved in the study design section while the worst was data collection section.

Moreover, the overall score ranged between 83 [17] and 104 [16] (Table 2). The difference between the scores achieved by the two studies conducted by Miners et al. [14, 15] depends mainly on the lack of a valid justification about the choice of the economic analysis form (first section) and the lack of indirect benefits/productivity changes (second section). These issues were included in the study published in 2002 [14] but were not analysed in the updated evaluation [15].

According to Drummond's checklist [11], modified by La Torre et al. [12], 60% were high quality studies and 40% were medium quality, mainly because methods and data collection were not exhaustively described.

## 4. Discussion

The aim of this review was to examine the methodological aspects and results of published economic evaluations on haemophilia A available treatments from 2002 to 2014.

The authors of the reviewed studies strongly agreed on the fact that prophylaxis, compared to on-demand treatment, achieves better health outcomes at additional costs, despite the different methodological approaches.

The extreme variability among the results of the studies is essentially due to several factors: first, the different time horizon used in all the analyses, and, secondly, the discount rate that can affect the results, especially if it is not applied to both costs and benefits. Other important factors that should be considered are

- the method that follows collect data (i.e., trials and cohort studies);
- unit costs of clotting FVIII;
- assumptions about utility.

In this regard, Lippert and colleagues' results [17] should be analysed as significantly different from those in other studies. This depends mainly on the short time horizon that is not sufficient to take into account long-term effects. In a time horizon of only one year applied in their analysis, the QUALYs gained are surely minimal (from 0.0187 to 0.0586 QALY for the different cases) with high ICERs (€1.2 and 5.7 million/QUALY) compared to a lifetime model [17].

In the Italian context, the analysis carried out by Capri and Ricciardi in 2011 as part of a Health-technology assessment (HTA) [19] also underlines the cost-effectiveness of primary prophylaxis with FVIII compared to on-demand therapy. They found an ICER of €35.036 in a time horizon of 70 years. The ICER obtained by Capri and Ricciardi [19] is less than €40.229 achieved by Colombo et al. for primary prophylaxis in a lifetime model [18]. Both studies were conducted from the perspective of the Italian National Health System (NHS) in the same year and due to lack of suitable Italian data, the assumptions about utilities were derived from Miners et al. 1999 [20].

The results of the two Italian analyses differ principally for the unit cost of factor therapy (€0.75 for Capri and Ricciardi versus €0.68 for Colombo et al.), the time horizon, and for the difference in FVIII half-life which influences the quantity of clotting factor required. Hence, besides the cost of treatment, an economic evaluation should also take into account the immunogenic profile of the factor therapy, which was not considered in the study by Colombo et al. [18].

The only non-European economic evaluation reviewed is the Canadian study [16], which compared standard prophylaxis and escalating-dose versus on-demand therapy over 5 years. The ICERs found (€379.134 and €698.300) are almost a tenth of the value reported by European studies. Also in this case, the variability of the results can be explained with the different time horizon, treatment regiments, and quantity/costs of clotting factor considered.

The studies reviewed can be also compared, for some aspects, to the recent cost-utility analysis of severe haemophilia A treatment over a lifetime horizon performed by Farrugia et al. 2013 [21]. Their model reflects the importance of inhibitor development and included the use of low-dose tolerization protocol in the first years of life as soon as a bleeding tendency manifests and then changed to the childhood and adult pharmacokinetic schedules. This approach is associated with a low probability of high cost inhibitor therapy as reported by Kurnik et al. 2010 [22] and results in the dominance of prophylaxis over on-demand treatment.

The model by Farrugia et al. [21] was influenced mainly by the dosage/cost of FVIII and inhibitor development. In particular, prophylaxis was dominant over on-demand treatment in UK due to the low cost of FVIII. In the USA case, prophylaxis has a higher cost over on-demand therapy due to the high cost of FVIII, but the ICER of $68.000 (€54.339 (Exchange rate $1 = €0.7987 [Updated to November 4, 2014])) is still within the range that is considered justified and is similar to European values [14, 15, 18].

Finally, in Sweden, the ICER was SEK 879.168 (€94.598 (Exchange rate 1SEK = € 0.1076 [updated to November 4, 2014])) over a life span [21], which is clearly less than the findings by Lippert et al. [17] of 5.7 million/QUALY in a year for patients with severe haemophilia.

According to the Guidelines for haemophilia, one option for the treatment of very young children is to start prophylaxis once a week [23] and the initiation of prophylaxis from an early age can obviate bleeding complications [6, 23–25]. Thus, the model proposed by Farrugia et al. [21] has strong basis, but more clinical evidence is needed to increase the significance of the low-dose regimen since the assumptions were derived from a pilot study and the optimal regimen remains to be defined.

Considering the quality assessment, the less discussed aspects in the reviewed studies (tackled in only one paper out of 5) are the details of the subjects from whom health states evaluations and justification about the choice of the discount rate were obtained: aspects that are present in the work by Farrugia et al. [21].

A limit of the present review is surely the period limitation for the studies (2002–2014) that excludes analyses performed in previous years. On the other hand, the specified period gave us the possibility to concentrate on the results of recent publications. The reviewed articles were then compared to other works, including the most recent. A particular attention is also dedicated to the Italian setting, considering the origin of most authors of the present work. These aspects differentiate our review from the work by Miners [26], which included cost-effectiveness, cost-benefit, and cost-utility analysis up to 2012.

## 5. Conclusion

The reviewed studies highlight the undoubted benefits of prophylaxis, as reported in the Guidelines for the Treatment of Hemophilia [23]. Yet some aspects of the economic models proposed might be further developed. Specifically, full economic analysis of haemophilia treatment should also consider the cost therapy in presence of inhibitors, which can affect ICER significantly: these issues were not addressed in the studies included in the review.

The recent work by Farrugia et al. [21] presents an integrated clinical scenario compared to previous analyses for a lifetime therapy [14, 15, 18]. The new model proposed could be the response to the bleeding problems in the increasing population of aged hemophiliacs, since early low-dose prophylactic protocol decreases the inhibitor incidence and bleeding complications, underlining the dominance of prophylaxis over on-demand treatment.

In conclusion, the cost of treatment, inhibitor development, and the immunogenic profile of the factor therapy should be considered in economic analyses to obtain a model that is closer to clinical reality, cost, and consumption of resources in each country. In fact, in most of the evaluations, assumptions are derived from the literature due to the lack of current data, as in the Italian case. We suggest conducting further economic evaluations in the Italian context in order to provide clear and specific data for the NHS and to obtain more accurate results for the treatment of haemophilia A.

## Figures and Tables

**Table 1:** Characteristics of the included studies.

Reference	Type of analysis	Alternative	Country/perspective	Patients/time horizon	Effectiveness measurement/cost measurement	Results
Miners et al., 2002 [14]	Cost-utility analysis	Primary prophylaxis versus on-demand with clotting factors	UK/societal	Hypothetical cohort of 100 patients with severe haemophilia A o B/severe Von Willebrand disease Time horizon: lifetime	QALYs/direct medical costs and indirect costs	**QALY**: primary prophylaxis 55.89 versus on-demand 41.10 **ICER**: *£*46.500(€59.315^a^)/QALY **Primary prophylaxis is more cost-effective than on-demand therapy**

Lippert et al., 2005 [17]	Cost-utility analysis	Secondary prophylaxis versus on demand	Germany, Sweden, UK, and The Netherlands/third party payer	Patients with severe haemophilia A and B without inhibitor (age ≥ 14 years) Time horizon: one year	QALYs and avoided bleeding episodes/direct medical costs	**ICER**: patients ≤ 30 years old and HIV positive €1.2 million/QALY (Germany); €1.72 million/QALY (UK) Patients ≤ 30 years old and HIV negative €2.21 million/QALY (Germany); €3.10 million/QALY (UK) Patients > 30 years old and HIV negative €4.77 million/QALY (Germany) €5.7 million/QALY (Sweden, UK) **Prophylaxis is more effective than on-demand treatment, but it causes higher costs**

Risebrough et al., 2008 [16]	Cost-utility analysis	EscDose versus on-demand Primary prophylaxis versus EscDose	Canada/societal	Individuals with severe haemophilia A, between 1 and 6 years old Time horizon: 5 years	QALYs and joint bleeding events avoided/direct medical costs and indirect costs (work days lost by parents)	**EscDose versus on demand:** significant reduction of bleeding (bleeding episodes decreased by 52 joint-bleeds when EscDose is compared to on-demand therapy) **ICER**: Can$542.938 (€379.134^b^)/QALY. **EscDose versusprimary prophylaxis ** **ICER**: >Can$1.000.000 (€698.300^b^)/QALY. **Prophylaxis can improve the quality of life of patients with haemophilia, though with high costs**

Miners, 2009 [15]	Cost-utility analysis	Primary prophylaxis versus on-demand with FVIII	UK/NHS	Hypothetical cohort of patients with severe haemophilia type A Time horizon: lifetime	QALY/direct medical costs	**QALY:** Primary prophylaxis 19.58 versus on-demand 13.95. Primary prophylaxis versus on-demand **ICER**: *£*38,000 (€48.469^a^)/QALY **Primary prophylaxis is cost-effective compared with on demand therapy.**

Colombo et al., 2011 [18]	Cost-utility analysis	On-demand treatment versus (1) primary prophylaxis; (2) secondary prophylaxis; (3) hybrid regimen (primary prophylaxis followed by on-demand)	Italy/NHS	Hypothetical cohort of patients with severe haemophilia A Time horizon: lifetime	QALY/direct medical costs	**ICER** on-demand versus Primary prophylaxis: €40.236/QALY; Secondary prophylaxis: €40.229/QALY; Hybrid regimen: €119.134/QALY. **Primary and secondary prophylaxes are more cost-effective than the hybrid regimen.**

^a^Exchange rate *£*1/€1.2758, updated to November 4, 2014. ^b^Exchange rate Can$ 1/€0.6983, updated to November 4, 2014.

**Table 2:** Results of the quality assessment with scores^*^.

Economic evaluations analyzed	Miners et al., 2002 [14]	Lippert et al., 2005 [17]	Risebrough et al., 2008 [16]	Miners, 2009 [15]	Colombo et al., 2011 [18]
Study design (maximum score 26)	26	23	26	23	19
Data collection (maximum score 45)	34	28	39	29	29
Analysis and interpretation of results (maximum score 48)	39	32	39	39	36
Total (maximum score 119)	**100**	**83**	**104**	**91**	**84**

^*^According to Drummond's checklist modified by La Torre et al. [12].
